# Effects of wearing a surgical face mask on cognitive functioning and mood states: a randomised controlled trial in young adults

**DOI:** 10.1007/s10339-024-01238-5

**Published:** 2024-10-23

**Authors:** Neda Nasrollahi, Tim Jowett, Liana Machado

**Affiliations:** 1 Department of Psychology and Brain Health Research Centre, University of Otago, William James Building, 275 Leith Walk, Dunedin, 9016 New Zealand; 2 Department of Mathematics and Statistics, University of Otago, Dunedin, New Zealand

**Keywords:** COVID-19, Cognition, Affect, Anxious, Crossover

## Abstract

Despite significant public concerns voiced about wearing face masks and reports from healthcare workers of adverse effects on cognition, research into potential adverse effects remains limited. The present trial investigated the effects of wearing a surgical face mask for prolonged periods on cognitive functioning and mood. We tested 42 university students (18–36 years old) using a controlled counterbalanced crossover design that involved a mask session and a control session, separated by 1 week. The two sessions were identical except that on the day of the mask session, participants were asked to wear a surgical mask for at least 8 h and to continue wearing it while visiting our laboratory, during which cognitive performance and mood were assessed as per the control session. Results showed that participants reported feeling less happy and more tense during the mask session compared to no-mask control. Additionally, cognitive performance differed between the two sessions for a selective attention task, reflecting slower response latencies during the mask session, which for the most part appeared to be driven by those who felt anxious wearing the mask. Although significant differences emerged for only two of six mood scales and one of eight cognitive tests, the evidence of adverse effects in a university population signals a need for research investigating vulnerable populations. Individuals with elevated anxiety may be particularly important to target.

*Trail Registration*: This randomized controlled trial was retrospectively registered (ACTRN12620001215910). Date registered: 16/11/2020, retrospectively registered.

**Supplementary Information:**

The online version contains supplementary material available at 10.1007/s10339-024-01238-5.

## Introduction

Following the Centre for Disease Control and Prevention (CDC) and World Health Organization (WHO) recommendations of wearing face masks in public to slow the spread of severe acute respiratory syndrome coronavirus (SARS-CoV-2) during the COVID-19 pandemic that commenced in 2019, the number of people wearing face masks increased universally, particularly in countries with mandatory regulations. While wearing a face mask reduces viral transmission, expectations that people wear a face mask have triggered significant controversy, sparking mass protests around the world. One of the most significant public concerns relates to fears that the use of face masks can adversely affect the brain.

Currently, very little is known about the cognitive and mood effects of wearing the types of face masks commonly worn during the COVID-19 pandemic. Past studies investigating the effects of wearing face masks on cognitive functioning have focused on industry-relevant respirators (i.e., dust, powered-air purifying, full-face negative-pressure respirators), rather than the types of face masks adopted by the public (e.g., disposable surgical masks or fabric face coverings). For instance, the results of a study by AlGhamri et al. (2013) showed an adverse effect of wearing a respirator on cognitive performance (as evidenced by an increased error rate) only for one of the respirators included in their study (full-face negative pressure respirator). Caretti (1999) reported that the overall effect of wearing a U.S Army full-face air-purifying respirator on cognitive performance was minimal, however, there was some adverse effect of wearing low resistance respirators on some cognitive abilities (as evidenced by slower response speed and lower response throughput for a serial addition/subtraction task). In addition, they reported no significant effects of wearing the respirators on six mood and behaviour factors (activity, anger, depression, fear, happiness, and fatigue).

In a recent survey by Rosner (2020), among 343 healthcare professionals, 23.6% reported impaired cognitive performance due to the prolonged use (typically a full work shift) of surgical and N95 masks. Following up on this, the results of a study by Morris et al. (2020) that objectively measured cognitive effects of wearing N95 masks for 45 min revealed a medium sized, albeit non-significant, effect on cognitive performance in the direction of worse performance during face mask use. However, it should be noted that their study was conducted on a very restricted sample (8 males). Insufficient power could potentially underpin the lack of significant effects. Moreover, the generalizability of their findings to the public is questionable due to all participants being male and their use of N95 masks, which were not recommended for use by the general public during the COVID-19 pandemic in relation to CDC (Updated 2021b) recommending that N95 masks should be prioritized for use in healthcare settings.

Consistent with Morris et al. (2020), another recent randomised controlled trial (*n* = 44) showed a small non-significant adverse effect of wearing N95 tight-fitting filtering facepiece respirator (FFR) masks (worn for just 15 min) on response time compared to a no mask condition in adults (Spang and Pieper 2021). Moreover, the results of a study (*n* = 133) by Schlegtendal et al. (2022) found no significant effect of wearing FFP2/K95 or surgical masks for two school lessons on cognitive performance of children. In line with this, a study by van Kampen et al. (2023) did not show any adverse effect of wearing face masks (surgical, cloth or FFP2) for 4 h on cognitive performance. In contrast, Smerdon (2022) showed that wearing a face mask (type not specified) initially reduced cognitive performance of competitive chess players; however, the effect was short-lived, as evidenced by no measurable adverse effect after ~ 4 h of play, which they proposed might reflect adaptation.

To address the limited objective evidence to date pertaining to prolonged use of face masks, the present study involved a randomised-controlled trial investigating the effects of wearing a disposable surgical mask on cognitive performance and mood. We used a counterbalanced crossover design with a 1-week washout. We decided to investigate surgical masks, as opposed to N95 masks, as these were recommended by CDC (Updated 2021a) for use by members of the public during the COVID-19 pandemic (although it should be noted that surgical masks were not necessarily the most common mask type; e.g., see Yeung et al. 2023). The intervention involved asking participants to wear a surgical face mask for at least 8 h, given that the mask mandates in many countries that required people to wear a face mask while in public led much of the public to wear a mask throughout a workday (but note that mask mandates did not come into effect in New Zealand until after this trial ended).

The current study tested the hypothesis that wearing a surgical face mask for an extended period would adversely impact cognitive functioning and mood, as evidenced by poorer cognitive performance and worse mood profiles (i.e., less positive and more negative). Findings reviewed in the preceding paragraphs suggest that our hypothesis in relation to cognitive performance may be supported. Although most of the reviewed studies were conducted under acute conditions, and thus the effects would not necessarily generalise to prolonged conditions, the self-reported impaired cognitive performance reported in the study by Rosner (2020) reflected prolonged face mask use. Regarding the predicted negative shift in mood profiles, although to our knowledge no studies to date have reported on the effects of wearing a surgical face mask on mood, the combination of the negative responses to requirements to wear a face mask from many members of the public and the cognitive difficulties self-reported by healthcare workers suggest that mood might be adversely affected.

## Method

This study was approved by (D19/297) and participants provided written informed consent prior to participating. Data collection began in July 2020 and ended in October 2020 (but note that there was a 2 month break due to COVID-19 related government restrictions and that there were no mask mandates in place in the region during data collection). This randomised controlled trial was retrospectively registered (ACTRN12620001215910).

## Overview

**Fig. 1:**
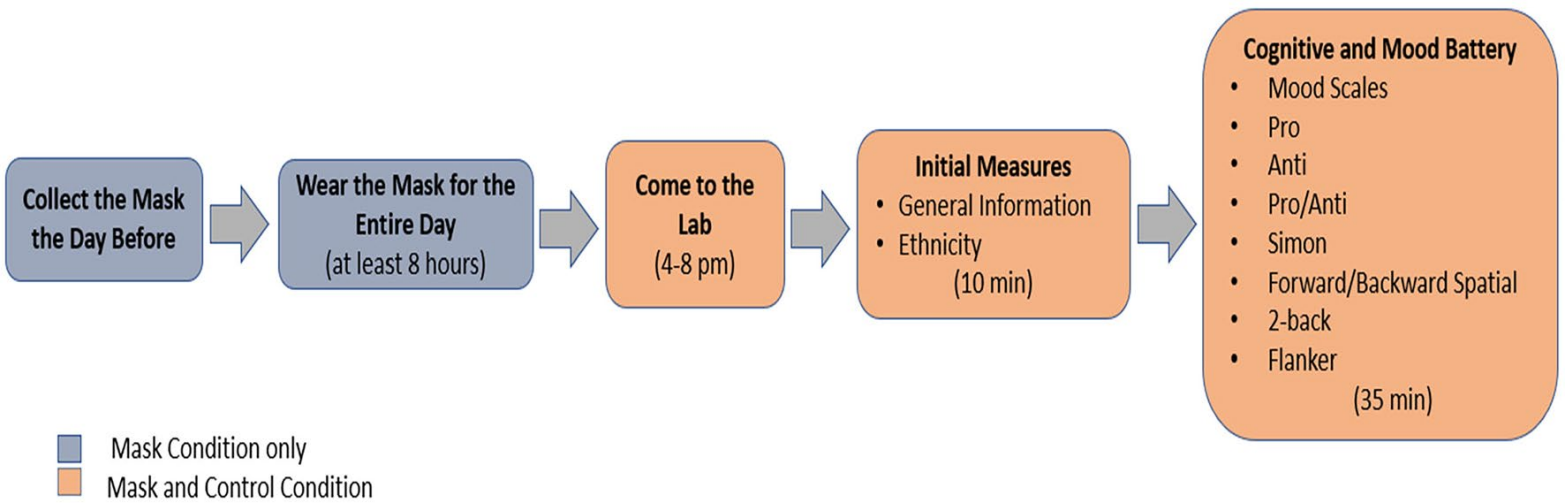
A step-by-step outline of the procedure

The present study used a randomised controlled crossover design with session order (mask or control session first) counterbalanced across participants. We used a prepopulated log sheet on Microsoft Excel to generate randomised session order. To maintain counterbalancing, participants who did not show up for their second session were replaced (as it became clear that each would be excluded). Participants visited our laboratory on two occasions at the same time of the day, exactly 1 week apart. Due to the nature of the intervention, the experimenter and participants were not blinded. A reminder was sent to participants the day before each session. After arriving at the laboratory for the first session, participants were asked to complete a general information questionnaire asking about age, years of education, sex, dominant hand, normal or corrected to normal vision and colour blindness, physical health, any respiratory related conditions (e.g., asthma), medication intake for any neurological or psychological disorder, and whether they felt comfortable to wear the mask. They were then given an ethnicity questionnaire to complete that included the ethnicity question from the New Zealand (NZ) census, after which they completed cognitive and mood testing. Each session took approximately 45 min to complete. Figure 1 provides an overview of the procedure.

## Participants

**Fig. 2:**
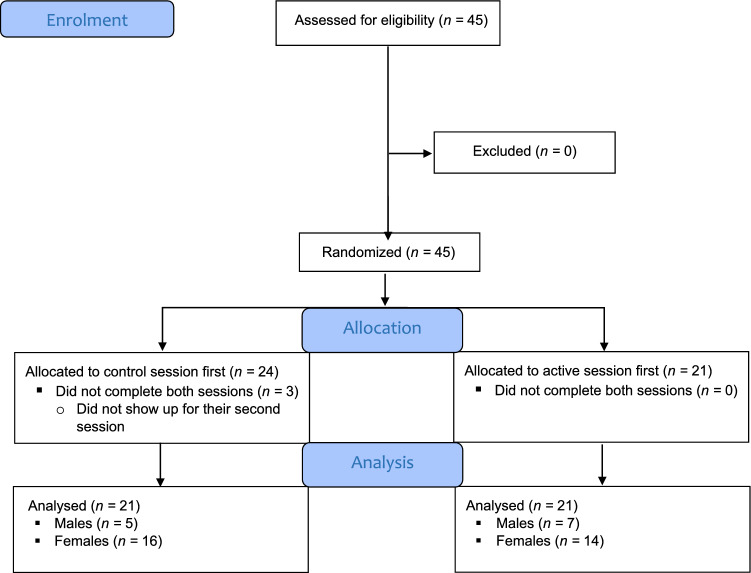
Flow-chart of participant recruitment and progression *Note* The final sample size of 42 was reduced for two of the cognitive measures: 2-Back (*n* = 38) and Backward Spatial (*n* = 40)

We initially recruited 45 undergraduate students from the Department of Psychology at the University of Otago in association with a course (note that some were students of the investigators). Inclusion criteria were as follows: aged at least 18 years, normal or corrected to normal vision, no history of chronic obstructive pulmonary disease, not feeling unwell (as required by the COVID-19 guidelines in place at the time) and willing to wear a face mask for an entire day. Data from three participants had to be excluded because they did not show up for their second session for the following reasons: one participant was no longer willing to wear the mask; one participant was feeling unwell on the day of participation and one participant for an unknown reason. Thus, the final sample included in the analyses comprised 42 young adults (*M*_age_ = 20.21 years, *SD* = 2.90, range = 18–36; 30 females, 40 right-handed; see Fig. 2 for a summary of participant recruitment and progression through the trial). The ethnicity groups reported by participants were: NZ European (*n* = 27), Asian (*n* = 4), Indian (*n* = 4), NZ European/Māori (*n* = 2), Middle Eastern (*n* = 2), Māori (*n* = 1), Samoan (*n* = 1) and British (*n* = 1).

## Procedure

### Mask session

Participants came to the laboratory to collect their mask the day before the mask session and were given instructions to wear the mask (Safe Plus anti-fog, earloop type, manufactured by BH medical products Co., Ltd.) for at least 8 h throughout the day of testing and continue to wear it until asked to remove it at the end of the testing session. It should be noted that the instructed period of mask wearing (at least 8 h) included break times that they were told to pull the mask off for eating, drinking and exercise, but not for more than 30 min in each break. On the day of participation, they visited the laboratory at their assigned time in the late afternoon (4–8 pm) while they still had their mask on and completed cognitive and mood tests. At the end of the session, they were asked to remove the mask and rate how anxious wearing the mask made them feel, on a Likert scale ranging from 1 (not at all) to 9 (extremely anxious).

### Control session

All aspects of the control session were identical to the mask wearing session, except that participants did not collect or wear a mask during the day of participation including during cognitive and mood testing.

## Cognitive and mood battery

The cognitive and mood battery, which took approximately 35 min to complete, was computerised and designed to assess current mood states and a variety of cognitive functions relevant to daily activities: basic visuomotor performance (Pro), inhibitory control (Anti), mental flexibility (Pro/Anti), selective attention (Simon, Flanker), and short-term (Forward Spatial) and working (Backward Spatial, 2-back) memory. The battery was programmed in MATLAB R2016b (The Mathworks, Natick, MA) and the Psychophysics Toolbox3 (Brainard 1997; Pelli 1997) and presented on a computer monitor with a black background positioned at a viewing distance of approximately 57 cm.

The battery has been used in previous randomised controlled trials (Forsyth et al. 2016; White et al. 2020) and assessed for test–retest reliability in a similar population (White et al. 2018). The tests were presented in the order described in the subsections that follow. We decided to present the mood scales before the cognitive tests to prevent potential fatigue from completing the cognitive tests contaminating the mood scale ratings. Participants were given verbal and written instruction at the start of each.

### Visual analogue mood scales (VAMS)

These scales were used to assess current mood state for the following six moods: sad, energetic, tense, happy, tired, and calm. For each mood scale, the instructions “click the position on the line that best represents how you feel right now” appeared along with a 100 mm horizontal line with a descriptor at each end: “not at all” on the left and “extremely” on the right. Participants clicked on the line, which caused a vertical line to appear, indicating their response; they could readjust the position of their response until they pressed a button labelled done. The line had 101 clickable positions. The most leftward point on the line was associated with a score of 0 and the most rightward point on the line was associated with a score of 100. For further details, refer to Machado et al. (2019).

### Pro, anti, and pro/anti

In these tasks, which have been reported previously (e.g., Cameron et al. 2015; Guiney et al. 2015), participants were asked to focus on a 0.3 cm white fixation dot until (after a variable interval: 400, 600, 800, 1000 or 1200 ms) a 2 cm green or red square appeared 8 cm to the left or right of the fixation dot (measured centre to centre). During Pro, participants were asked to press the button on the same side as the square, which was always green. During Anti, participants were asked to press the button on the opposite side of the square, which was always red. During Pro/Anti, the square was green or red at random and participants were asked to press the button on the same side when green and on the opposite side when red. Participants completed four practice trials followed by 40 test trials each for Pro and Anti, and six practice trials followed by 40 test trials for Pro/Anti. Note that for the speeded tasks (i.e., Pro, Anti, Pro/Anti, Simon and Flanker), we pre-randomized the trial sequences and then used a fixed trial sequence for each to avoid variation related to particular trial sequences; all conditions and condition combinations occurred equally often; participants were asked to respond as quickly as they could without compromising accuracy using the index finger of each hand to press the left or right button on a button box (DirectIN Rotary Controller, Empirisoft Corporation, NY); a 900 Hz error tone sounded for 300 ms if the participants made an incorrect response, made no response within 1500 ms, or responded in less than 100 ms; and between trials the screen turned black for either 500 ms (Pro, Anti, and Pro/Anti) or 1000 ms (Simon, Flanker).

### Simon

This task, which we adapted from Kouwenhoven and Machado (2024), assessed the ability to ignore irrelevant spatial information. For this task and the flanker task, two consonants (C and T) were randomly selected and assigned to each of the response buttons prior to the start of this intervention (this mapping was used in all cases). Participants were instructed to press the left button if C appears and the right button if T appears. After a variable fixation period (400, 600, 800, 1000 or 1200 ms), one of the two consonants appeared positioned 3 cm to the left or right of centre (measured centre to centre), which varied randomly with respect to the side of the correct response button (left or right), such that they were either spatially compatible or incompatible. Participants completed six practice trials followed by 60 test trials.

### Forward/backward spatial

For these tasks, which were adapted from the Corsi Block Tapping task (Corsi 1972), nine grey boxes (3.2 cm wide × 3.0 cm high) appeared on the screen and after 500 ms, boxes turned white one at a time for 1000 ms (with no interstimulus interval) in a predetermined sequence (for further details see Table S1). At the end of each sequence, a 400 Hz tone sounded for 300 ms, and participants indicated the sequence using a mouse to click on boxes (in the order viewed for Forward Spatial, and in reverse order for Backward Spatial). Each sequence length occurred twice, beginning with a length of two boxes; if the participant responded correctly to at least one of the two, the program increased the length of the sequence by one box up to a maximum length of nine boxes; if not, the program ended and recorded the participant’s score, which was the product of the length of the last accurately recalled sequence and the number of sequences correctly recalled (maximum score = 144). Two practice sequences, each two boxes in length, preceded the test sequences.

### 2-back

This task assessed working memory. Participants were presented with a sequence of 90 consonants (excluding W, Y and Z). Each letter was displayed for 500 ms (with an interstimulus interval of 2500 ms), and participants indicated whether each letter matched the letter 2-back in the sequence (using the right button to indicate match and the left button to indicate no match). Responses to the first two letters were not recorded. A 900 Hz error tone sounded for 300 ms if the participants made an incorrect response, made no response within 3000 ms, or responded in less than 100 ms. Thirty letters matched the letter 2-back in the sequence. Three unmatched letters matched the letter 3-back in the sequence and another three unmatched letters matched the letter 1-back in the sequence. For further details, refer to White et al. (2018). A practice sequence consisting of 15 letters, which included five 2-back matches, preceded the test sequence.

### Flanker

This task, which we adapted from Machado et al. (2013), assessed the ability to ignore irrelevant identity-based information. After a variable fixation period (400, 600, 800, 1000 or 1200 ms), two letters (each randomly selected from the two possible letters: C and T) appeared simultaneously, one serving as the target (positioned at centre, replacing the fixation dot) and the other serving as a distractor (positioned above or below the target randomly, with 1 cm separating them). The distractor could be the same as the target (compatible) or the other letter (incompatible). Participants were instructed to respond to the central letter by pressing the assigned key while ignoring the distractor. Ten practice trials preceded 40 test trials.

## Statistical analysis

For Pro, Anti, Pro/Anti, Simon and Flanker, we calculated median reaction time (RT) in ms for correct responses and the percentage of correct responses. We used correct median RTs as the performance indicator because ceiling accuracy rates were anticipated based on past research using these cognitive tasks (White et al. 2018). For Forward and Backward Spatial, we calculated scores by multiplying the longest sequence length at which at least one sequence was recalled correctly by the total number of sequences recalled correctly (Kessels et al. 2000). For 2-back, we calculated overall accuracy for the 88 test letters associated with a response in accordance with past research (Forsyth et al. 2016).

To determine whether test/trial type influenced any session differences, generalised estimating equation (GEE) models (Liang and Zeger 1986) with session (mask, no-mask control) and test/trial type as within-subject factors, were conducted for all of the cognitive tasks. Because of the skewed nature of the outcome variables, it was deemed that conventional repeated measures ANOVA, which assumes normally distributed outcome variables, would not be optimal. A key advantage of GEE models, compared with repeated measures ANOVA, is that GEE models do not require distributional assumptions for the observations. Instead, the GEE models only require a regression model for the mean values (Fitzmaurice 2012; note that for the speeded tasks, mean of the median correct RT is modeled). As well as the primary effects of interest, the GEE models also included three VAMS covariates along with scores from the Likert scale enquiring about how anxious wearing the mask made them feel and whether they reported having neurological or psychological disorder. The three VAMS variables used in the models were: Calm, Happy and Tired. The other VAMS variables (Sad, Energetic, Tense) were excluded due to multicollinearity (correlation) issues with the VAMS included in the model. Within each task, a crossover experimental design (Puntanen 2008) was used to investigate and allow for the possibility of a carryover effect between sessions. To assess and adjust for a carryover effect, the models included the effects of “session order” (the order in which each participant carried out the sessions) and “test period” (whether a session was the first or second session attended for each participant).

For the main purpose of the study as specified in our trial registration, paired-sample *t* tests were conducted to examine differences in mood scale ratings and cognitive performance between the two sessions. A priori power analysis using G*Power version 3.1 (Faul et al. 2007) indicated that a sample size of 40 participants would be required to achieve 80% power using paired-sample *t* tests to detect an approximately medium-sized effect of wearing a face mask on cognitive functioning (Hedges’*g* ~ 0.46 corresponding to η^2^ ~ 0.05), which could be expected based on the reported effect of wearing face masks on math-motor performance (Morris et al. 2020). However, it should be noted that some of the effect sizes reported by Morris et al. (2020) were much smaller (Hedges’*g* ~ 0.13 corresponding to η^2^ ~ 0.004), below the level that our study was powered to detect. In an effort to detect smaller effect sizes, we originally aimed to test more participants, however this was disrupted by government imposed restrictions during the testing phase of this trial, which prevented data collection for a period of 2 months, and once restrictions had lightened sufficiently to enable data collection, potential volunteers were likely deterred. The interpretation of effect sizes in the current study was based on recommendations in Cohen (1988): a small effect (Hedges’ *g* ~ 0.20, *r* ~ 0.10 and ƞ_p_^2^ ~ 0.01), a medium effect (Hedges’ *g* ~ 0.50, *r* ~ 0.30 and ƞ_p_^2^ ~ 0.06), and a large effect (Hedges’ *g* ~ 0.80,* r* ~ 0.50 and ƞ_p_^2^ ~ 0.14). We used *p* < 0.05 as the significance level and *p* < 0.1 as trend level and the threshold for interpreting our results. All analyses were conducted using IBM SPSS (version 25.0) except for the GEE models, which were conducted using R (version 4.0.2).

Materials and data for this study are available by emailing the corresponding author.

## Result

### Participant characteristics

Table 1 summarizes participant characteristics. Participants reported wearing the face mask on average for 7.9 (range = 4–11, SD = 1.12) hours before arriving at the mask wearing session and all participants also reported not wearing a mask on the day of the no-mask control session (note that there were no government-imposed mask mandates in place during data collection).

**Table 1:** Participant characteristics (n = 42)

Variables	*M* (Range)	*n* (%)
Age (years)	20.21(18–36)	
Gender (female)		30 (71.43)
Handedness (right)		40 (95.24)
Chronic obstructive pulmonary disease		0 (0.00)
Asthma		7 (16.70)
Neurological or psychological disorder		13 (30.95)
How anxious wearing the mask made participants feel	4.98 (1–9)	

How anxious wearing the mask made participants feel is on a Likert scale ranging from 1 (not at all) to 9 (extremely anxious)

## Mood states

The descriptive statistics for the VAMS in each session are presented in Table 2 and show that in all cases ratings were in the predicted direction of less positive (energetic, happy, calm) and more negative (sad, tense, tired) during the mask session relative to the no-mask control session. Paired-sample *t* tests comparing mood scale ratings between the control and mask sessions revealed significant small-to-medium-sized effects for happy, *t*(41) = − 2.53, *p* = 0.015, *g* = 0.41, and tense, *t*(41) = 2.12, *p* = 0.040, *g* = 0.30, indicating that participants reported feeling less happy and more tense during the mask compared to the control session. In addition, feelings of sadness showed a fairly small effect size in the direction of more sad during the mask session that only reached trend level, *t*(41) = 1.86, *p* = 0.070, *g* = 0.31. None of the other mood state differences between the two sessions met our threshold for interpretation, *t*(41) ≤ 1.12, *p* ≥ 0.124, *g* ≤ 0.27, in all cases.

**Table 2:** Summary of cognitive performance and mood states in the control and mask wearing sessions (n = 42)

	Control		Mask		Session Difference		
	*M*	*SD*	*M*	*SD*	*95% CI*	*g*	%
*Mood States*							
Sad	13.14	14.36	18.14	16.94	[− 0.42, 10.42]	0.31	38.05
Energetic	47.81	20.54	45.33	18.41	[− 10.60, 5.64]	0.12	− 5.18
Tense*	27.00	20.16	33.16	20.13	[0.30, 12.03]	0.30	22.81
Happy*	65.69	14.51	59.69	14.43	[− 10.80, − 1.21]	0.41	− 9.13
Tired	53.66	19.78	57.95	20.44	[− 3.43, 12.00]	0.21	7.99
Calm	64.83	17.49	59.62	19.86	[− 11.91, 1.50]	0.27	− 8.04
*Cognitive Tests*							
**Pro**							
Median RT (ms)	298	37.65	307	43.32	[− 2.00, 20.00]	0.22	3.02
Accuracy (%)	99.70	0.98	99.70	0.82	[− 0.30, 0.30]	0.00	0.00
*Anti*							
Median RT (ms)	341	41.35	353	52.66	[− 2, 30.00]	0.26	3.52
Accuracy (%)	98.57	1.84	98.57	2.08	[− 1.00, 1.00]	0.00	0.00
*Pro/Anti*							
Median RT (ms)	479	67.41	482	77.35	[− 2.00, 30.00]	0.04	0.63
Accuracy (%)	96.37	3.32	95.36	4.40	[− 3.00, 0.40]	0.25	− 1.05
*Simon compatible*							
Median RT (ms)	432	41.44	434	60.07	[− 14.00, 20.00]	0.05	0.46
Accuracy (%)	97.78	2.91	98.17	2.47	[− 1.00, 1.40]	0.14	0.40
*Simon incompatible*							
Median RT (ms)	473	48.10	486	59.55	[− 10.00, 32.00]	0.24	2.75
Accuracy (%)	95.24	5.11	94.52	6.45	[− 3.00, 1.40]	0.12	− 0.76
*Flanker compatible*							
Median RT (ms)	437	67.29	451	66.82	[− 1.00, 30.00]	0.21	3.20
Accuracy (%)	96.67	4.37	97.02	4.28	[− 2.00, 2.20]	0.07	0.36
*Flanker incompatible*							
Median RT (ms)**	463	72.95	488	73.96	[10.00, 40.00]	0.33	5.40
Accuracy (%)	96.31	5.19	96.90	3.82	[− 1.00, 2.20]	0.13	0.61
*Forward spatial*							
Score	67.42	22.77	63.66	17.48	[− 11.17, 3.65]	0.18	− 5.58
*Backward spatial*							
Score (*n* = 40)	63.52	26.26	61.00	25.47	[− 11.33, 4.24]	0.09	− 3.97
*2-Back*							
Accuracy (%; *n* = 38)	86.78	6.81	86.06	6.03	[− 6.60, 5.12]	0.11	− 0.83

*RT* Reaction Times, Backward Spatial and 2-Back had reduced sample sizes (see text for details). *p < .05, **p < .01. *g* = Hedge’s *g*; interpretation benchmarks: ~ 0.20 small effect, ~ 0.50 medium effect. *CI* confidence interval. %Difference = [(Mask-Control)/Control]*100

## Cognitive tests

Descriptive statistics for each cognitive variable for the control and mask sessions are displayed in Table 2. For one participant, data for Backward Spatial was missing due to technical difficulties and then to maintain our counterbalanced design of session order (in relation to potential practice effects that could lead to misleading results; White et al. 2018), data for one additional participant (the next participant with the opposite session order) had to be excluded (thus two participants excluded in total). For 2-back, we had to exclude data for three participants as they did not meet the minimum accuracy level of 60%, which risks chance performance (White et al. 2018). To maintain counterbalancing, we excluded 2-back data for one additional participant (the next participant following the third exclusion with the opposite session order, thus four participants excluded in total). As expected based on previous studies using the same cognitive tests (Forsyth et al. 2016; White et al. 2018), mean accuracy approached ceiling (≥ 94%) for all speeded tasks in which participants were instructed to respond as quickly as they could without compromising accuracy (i.e., Pro, Anti, Pro/Anti, Simon and Flanker) and paired-sample *t* tests confirmed no differences in mean accuracy between the control and mask sessions, *t*(41) ≤ 0.78, *p* ≥ 0.181, *g* ≤ 0.25 in all cases.

Regarding correct median RTs (the preregistered outcome variable), a 2 × 3 GEE model with session (mask, no-mask control) and test type (Pro, Anti, Pro/Anti) as within-subject factors showed, a main effect of test type, *χ*^*2*^(2) = 802.40, *p* < 0.001, η_p_^2^ = 0.80, reflecting slower RTs as task difficulty increased, which was expected based on previous research (Bierre et al. 2017; Nasrollahi et al. 2021). However, the main effect of session, χ^2^(1) = 1.76, *p* = 0.185, η_p_^2^ < 0.01, and the interaction between session and test type, χ^2^(2) = 1.42, *p* = 0.491, η_p_^2^ < 0.01, did not approach significance.

A 2 × 2 GEE model analysing correct median RTs with session (mask, no-mask control) and trial type (Simon Compatible, Simon Incompatible) as within-subject factors revealed a main effect of trial type, *χ*^*2*^(1) = 79.56, *p* < 0.001, η_p_^2^ = 0.39, reflecting the typical finding of slower RTs on incompatible trials (Kouwenhoven & Machado 2024). However, neither the main effect of session, *χ*^*2*^(1) = 1.12, *p* = 0.290, η_p_^2^ < 0.01, nor the interaction between session and trial type, *χ*^*2*^(1) = 2.55, *p* = 0.111, η_p_^2^ = 0.02, were significant.

A 2 × 2 GEE model with session (mask, no-mask control) and trial type (Flanker Compatible, Flanker Incompatible) as within-subject factors showed a main effect of session, *χ*^*2*^(1) = 10.52, *p* = 0.001, η_p_^2^ = 0.08, reflecting slower RTs in the mask compared to no-mask control session, and a main effect of trial type, *χ*^*2*^(1) = 106.88, *p* < 0.001, η_p_^2^ = 0.47, reflecting the expected pattern of slower RTs on incompatible trials relative to compatible trials (Kouwenhoven & Machado 2024). There was also a main effect of test period, *χ*^*2*^(1) = 13.90, *p* < 0.001, η_p_^2^ = 0.10, due to faster RTs in the second session attended, quantified by an estimated first session attended minus second session attended difference in means of 24.8 ms (95% *CI*: 13.6, 36.0). The interaction between how anxious wearing the mask made them feel and session was also significant, χ^2^(1) = 4.84, *p* = 0.028, η_p_^2^ = 0.09. How anxious wearing the mask made them feel had no significant relationship with Flanker RT during the control session, whereas in the mask session there was a statistically significant effect, with each 1-unit increase in the anxious score associated with a 9.3-unit increase in Flanker RT (95% *CI*: 1.7, 16.9). The interaction can also be expressed in relation to the slower RTs in the mask session compared with the control session; at 1 on the Likert scale, mean RT slowing was only -2.0 (95% *CI* − 20.6, 16.7), *Z* = − 0.21, *p* = 1.000, Cohen’s *d* = − 0.05, whereas at 9 on the Likert scale, mean RT slowing was -37.0 (95% *CI* − 57.0, − 17.1), *Z* = − 3.63, *p* = 0.001, Cohen’s *d* = − 0.79. The interaction between session and trial type was not statistically significant, *χ*^*2*^(1) = 2.16, *p* = 0.142, η_p_^2^ = 0.02.

A 2 × 2 GEE model with session (mask, no-mask control) and test type (Forward Spatial, Backward Spatial) as within-subject factors revealed a significant effect of how anxious wearing the mask made them feel, *χ*^*2*^(1) = 8.41, *p* = 0.004, η_p_^2^ = 0.18. The relationship with spatial test scores had a negative trend such that a 1-unit increase in the Likert scale rating was associated with a mean reduction in the spatial test scores of 3.06 (95% *CI* − 5.1, − 1.6), irrespective of the session. The model did not reveal any other significant effects; of note the main effect of session, *χ*^*2*^(1) = 1.40, *p* = 0.237, η_p_^2^ = 0.01, and the interaction between session and test type, *χ*^*2*^(1) = 0.04, *p* = 0.841, η_p_^2^ < 0.01, were not statistically significant.

A GEE model with session (mask, no-mask control) as a within-subject factor for the 2-back test did not reveal any significant effects; of note, the main effect of session did not approach significance, *χ*^*2*^(1) = 0.23, *p* = 0.632, η_p_^2^ < 0.01.

**Fig. 3:**
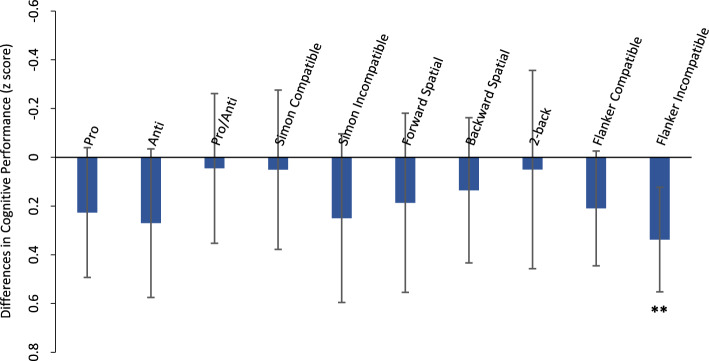
Cognitive Performance Difference (Mask vs. Control Session) for each Measure *Note* The variables used to calculate differences in cognitive performance were as follows: correct RTs in ms for Pro, Anti, Pro/Anti, Simon and Flanker; scores for Forward and Backward Spatial; and overall accuracy for 2-back. Sample size 42 except for 2-back (*n* = 38) and Backward Spatial (*n* = 40); see text for details about exclusions. Error bars represent 95% confidence interval. **p < .01

In addition to the GEE models, we computed the planned paired-sample *t* tests assessing differences in cognitive variables of interest between mask and no-mask control sessions, which revealed a significant but fairly small difference in cognitive performance between the two sessions for Flanker Incompatible trials, *t*(41) = 3.18, *p* = 0.003, *g* = 0.33, indicating 5.4% worse cognitive performance (as evidenced by slower RTs) during the mask session compared to the control session. It should be noted, however, that accuracy for Flanker Incompatible trials was slightly higher during the mask session, but this did not approach significance, *t*(41) = 0.74, *p* = 0.463, *g* = 0.13. Additionally, there were some non-significant trends (*p* < 0.1) for slightly worse cognitive performance during the mask session for Pro, Anti and Flanker Compatible trials, 1.72 ≤ *t*(41) ≤ 1.80, 0.080 < *p* < . 093, 0.20 ≤ *g* ≤ 0.27. None of the other cognitive performance comparisons between the two sessions met our threshold for interpretation, *t*(37–41) ≤ 1.50, *p* ≥ 0.152, *g* ≤ 0.24 in all cases. As the assumption of normality for Flanker Incompatible trials was violated (assessed by Shapiro–Wilk’s test, *p* < 0.05), Wilcoxon signed rank non-parametric tests were conducted. This confirmed the difference in cognitive performance between sessions for Flanker Incompatible trials, *Z* = − 2.90, *p* = 0.004, *r* = 0.44. As measured variables varied across the cognitive tests in the battery, we displayed differences in cognitive performance between the two sessions for each task using percent difference (see Table 2) and z scores (see Fig. 3).

## Discussion

The present study investigated the effects of wearing a surgical face mask on cognitive functioning and mood. To date, very few studies investigating the impact of wearing surgical face masks (which have been commonly worn in relation to the COVID-19 pandemic) have reported on cognitive functioning, and none have reported on mood. Consistent with our hypothesis, the results showed that wearing a surgical face mask had a small adverse effect on cognitive performance as evidenced by slower RTs during a selective attention task (Flanker), which mostly was driven by people who felt more anxious wearing the face mask. There were also some trends (*p* < 0.1) for slightly worse cognitive performance during the mask session for Pro and Anti. Furthermore, participants reported feeling less happy and more tense during the mask wearing session compared to the control session and there was a small trend towards feeling more sad during the mask session.

The finding of an adverse effect of wearing a surgical face mask on cognitive functioning in the current study is in line with the result of the recent survey by Rosner (2020), which provided self-report evidence that wearing surgical or N95 face masks for an extended period of time adversely affects cognitive performance. It should also be noted that although in the present study the adverse effect of wearing a surgical face mask on cognitive functioning only exhibited a statistically significant (*p* < 0.05) effect for one of the tasks (Flanker), the pattern of the data showed numerically worse performance for all cognitive tasks during the mask wearing session compared to the no-mask control session. In line with our findings, Smerdon (2022) found that wearing a face mask reduced cognitive performance of chess players, although interestingly there was no longer a measurable adverse effect after approximately 4 h of play, which suggested an adaptive response.

Although the findings of the present study seem to contrast with the result of the recent study by Morris et al. (2020), which found no adverse effect of wearing N95 face masks on cognitive functioning, the pattern of their data was in the direction of worse cognitive performance during face mask use, which is consistent with the pattern of data in the current study. Similarly, Caretti (1999) reported that the overall effect of wearing a U.S Army respirator on cognitive performance was minimal, however, there was some detrimental effect of wearing low resistance respirators on some cognitive abilities. Moreover, our pattern of data is consistent with the results of a recent study by Spang and Pieper (2021), which showed a small non-significant adverse effect of wearing N95 FFR masks for 15 min in adults ranging in age from 20 to 64 years. Similarly, although Schlegtendal et al. (2022) found no significant adverse cognitive effects of wearing FFP2/K95 and surgical masks during two school lessons, the authors acknowledged that there were some tendencies towards slightly worse cognitive performance in the mask wearing compared to no mask wearing groups. However, in contrast with our findings, van Kampen et al. (2023) did not detect any evidence of adverse effects of wearing face masks (surgical, cloth, or FFP2) for 4 h on cognitive performance; key factors behind the different results could relate to the amount of time the masks were worn and/or the particular cognitive functions tapped or sample assessed. Regarding the latter, whereas our sample was predominantly young women, their sample median age was 47 years (range = 19–65).

Regarding mood states, in contrast with the study by Caretti (1999) that reported no significant effect of wearing a U.S Army respirators on mood states, our participants reported feeling less happy and more tense and sad (although the latter was only trend level) during the mask wearing compared to the control session. This discrepancy may relate to the small sample size in Caretti (1999; n = 8). Alternatively, it has been suggested that breathing resistance caused by the mask materials may affect mood states (Caretti 1999). It might be the case that breathing resistance levels vary between the army respirators used in the study by Caretti (1999) and surgical masks used in the current study, which potentially may help explain the inconsistency between the two studies in findings related to mood states.

## Limitations and future research

Although in the current study we asked participants to wear the mask for at least 8 h, taking it off for breaks of no more than 30 min, some participants reported wearing the mask for as few as 4 h and we did not have any objective measure of how the masks were worn or what participants did during the time they were wearing the mask (e.g., rest or run errands). Moreover, some members of the public may take their mask off for periods longer than 30 min, reducing generalizability. Another limitation stems from the nature of the intervention preventing blinding. In addition, the cohort is not representative of the general population as the participants in the current trial were all university students (and consequently had a relationship with the investigators and likely had familiarity with computerized cognitive tests), mostly Aotearoa NZ European (64.28%) and mostly females (71.43%). Regarding the timing of our study, the fact that it took place fairly early on with respect to the progression of the COVID-19 pandemic in New Zealand on the one hand meant that participants did not feel compelled to wear a mask on the day of the control session, but on the other hand we suspect that the requirement to wear a mask may have put some people off the idea of participating and in the case of one participant unwillingness to wear the mask led to their exclusion from the study.

It should be noted that the current findings should not be assumed to generalize to other types of face masks commonly worn in public settings during the COVID-19 pandemic. As there are some potentially relevant differences between N95, surgical and cloth masks (e.g., different materials and facial fits), it would be beneficial to examine the comparative effects on cognitive functioning and mood. For instance, the results of recent studies by Marek et al. (2023) and van Kampen et al. (2023) showed that the effects of wearing a mask on subjective feelings pertaining to heat, humidity and difficulty breathing were stronger when wearing an FFP2 respirator than a surgical mask or cloth mask. It should also be noted that the current study had insufficient power to detect small effect sizes, which calls for caution in interpreting the results given that effects could be small in a healthy young adult population. Moreover, the cohort assessed in the current trial did not include a sufficient number of participants with potentially relevant health conditions (e.g., asthma, anxiety and depression), which may be an important avenue for future research as these populations may be more adversely affected by wearing face masks. On the other hand, some caution should be taken in interpreting the statistically significant results reported here given that we conducted multiple statistical tests without adjustment to reduce the risk of false positives (Type I errors). The reason for this methodological decision relates to the early-stage nature of the current research and a focus on identifying potentially interesting effects for further investigation. In this context, the risk of false rejections (Type II errors) would have been too high if we applied full adjustments for multiple testing.

## Conclusion

In summary, the current study revealed small-to-medium adverse effects of wearing a surgical face mask on cognitive functioning and mood characterised by a less positive mood profile and slower response latencies during a selective attention task (Flanker). The occurrence of significant effects for only two of the six mood scales and only one of the eight cognitive tasks suggests that wearing a surgical face mask for an extended period of time only had limited effects on cognition and mood. Based on the results, it seems that healthy young adults likely should not be too much impacted from wearing a surgical mask, with difficulties experienced most likely being relevant to selective attention and feeling more tense and less happy. The cognitive difficulties appeared to be driven largely by those who felt more anxious wearing the mask. This evidence of adverse effects in a university population signals that it may be beneficial to investigate the effects of wearing face masks in vulnerable populations such as older adults and people with pre-existing conditions such as asthma, anxiety or depression. In closing, the findings of this study suggest that when wearing a face mask to protect against virus transmission, taking sufficient breaks may be advisable especially in people who are more anxious.

## Supplementary Information

Below is the link to the electronic supplementary material.

Supplementary file 1 (DOCX 26 KB)

## Data Availability

Materials and data for this study are available by emailing the corresponding author.
