# Supplementary material for: Effects of wearing a surgical face mask on cognitive functioning and mood states: a randomised controlled trial in young adults
Source: Cogn Process. 2024 Oct 23;26(1):189–99. doi: 10.1007/s10339-024-01238-5 (PMC11897070; doi:10.1007/s10339-024-01238-5)
Supplement: Supplementary file 1 — (DOCX 26 KB) [file 10339_2024_1238_MOESM1_ESM.docx]

Title: Effects of Wearing a Surgical Face Mask on Cognitive Functioning and Mood States: A Randomized Controlled Trial in Young Adults

Neda Nasrollahi ^a^*, Tim Jowett ^b^, and Liana Machado ^a^

^a^ Department of Psychology and Brain Health Research Centre, University of Otago, Dunedin, New Zealand. ^b^ Department of Mathematics and Statistics, University of Otago, Dunedin, New Zealand. *Correspondence concerning this article should be addressed to Neda Nasrollahi, e-mail: [neda.nasrollahi@otago.ac.nz](mailto:neda.nasrollahi@otago.ac.nz).

**Table S1**

*Sequence Sets Used in Forward and Backward Spatial*

| Length | Forward Spatial | | | | | | | | |  | Backward Spatial | | | | | | | | | |
| --- | --- | --- | --- | --- | --- | --- | --- | --- | --- | --- | --- | --- | --- | --- | --- | --- | --- | --- | --- | --- |
| 2 | 5 | 7 |  |  |  |  |  |  |  |  | | 9 | 4 |  |  |  |  |  |  |  |
|  | 2 | 1 |  |  |  |  |  |  |  |  | | 7 | 5 |  |  |  |  |  |  |  |
| 3 | 4 | 3 | 6 |  |  |  |  |  |  |  | | 6 | 3 | 7 |  |  |  |  |  |  |
|  | 3 | 8 | 1 |  |  |  |  |  |  |  | | 2 | 1 | 8 |  |  |  |  |  |  |
| 4 | 6 | 1 | 5 | 8 |  |  |  |  |  |  | | 3 | 2 | 4 | 9 |  |  |  |  |  |
|  | 1 | 7 | 4 | 2 |  |  |  |  |  |  | | 6 | 1 | 5 | 8 |  |  |  |  |  |
| 5 | 9 | 4 | 7 | 2 | 1 |  |  |  |  |  | | 9 | 3 | 1 | 4 | 7 |  |  |  |  |
|  | 5 | 2 | 1 | 8 | 6 |  |  |  |  |  | | 6 | 2 | 8 | 1 | 3 |  |  |  |  |
| 6 | 7 | 9 | 2 | 5 | 1 | 3 |  |  |  |  | | 2 | 5 | 9 | 6 | 3 | 1 |  |  |  |
|  | 4 | 2 | 8 | 3 | 7 | 9 |  |  |  |  | | 5 | 6 | 8 | 1 | 9 | 4 |  |  |  |
| 7 | 5 | 4 | 1 | 6 | 3 | 9 | 7 |  |  |  | | 3 | 7 | 8 | 1 | 6 | 2 | 9 |  |  |
|  | 9 | 3 | 8 | 1 | 6 | 2 | 5 |  |  |  | | 8 | 1 | 7 | 3 | 2 | 4 | 5 |  |  |
| 8 | 1 | 4 | 7 | 2 | 8 | 3 | 6 | 9 |  |  | | 5 | 3 | 1 | 2 | 9 | 6 | 7 | 8 |  |
|  | 4 | 8 | 1 | 2 | 7 | 3 | 9 | 6 |  |  | | 3 | 8 | 7 | 1 | 2 | 4 | 6 | 9 |  |
| 9 | 3 | 7 | 4 | 1 | 9 | 2 | 5 | 6 | 8 |  | | 8 | 2 | 3 | 7 | 6 | 1 | 4 | 9 | 5 |
|  | 1 | 6 | 4 | 3 | 2 | 9 | 5 | 8 | 7 |  | | 3 | 6 | 7 | 4 | 1 | 5 | 9 | 2 | 8 |
